# Genetic Risk Score of *NOS* Gene Variants Associated with Myocardial Infarction Correlates with Coronary Incidence across Europe

**DOI:** 10.1371/journal.pone.0096504

**Published:** 2014-05-07

**Authors:** Robert Carreras-Torres, Suman Kundu, Daniela Zanetti, Esther Esteban, Marc Via, Pedro Moral

**Affiliations:** 1 Departament Biologia Animal – Antropologia, Universitat de Barcelona, Barcelona, Spain; 2 Department of Epidemiology, Erasmus University Medical Center, Rotterdam, The Netherlands; 3 Institut de Recerca de la Biodiversitat (IRBio), Universitat de Barcelona, Barcelona, Spain; 4 Departament Psiquiatria i Psicobiologia Clínica and Institute for Brain, Cognition and Behaviour (IR3C), Universitat de Barcelona, Barcelona, Spain; Universite de Montreal, Canada

## Abstract

Coronary artery disease (CAD) mortality and morbidity is present in the European continent in a four-fold gradient across populations, from the South (Spain and France) with the lowest CAD mortality, towards the North (Finland and UK). This observed gradient has not been fully explained by classical or single genetic risk factors, resulting in some cases in the so called Southern European or Mediterranean paradox. Here we approached population genetic risk estimates using genetic risk scores (GRS) constructed with single nucleotide polymorphisms (SNP) from nitric oxide synthases (*NOS*) genes. These SNPs appeared to be associated with myocardial infarction (MI) in 2165 cases and 2153 controls. The GRSs were computed in 34 general European populations. Although the contribution of these GRS was lower than 1% between cases and controls, the mean GRS per population was positively correlated with coronary incidence explaining 65–85% of the variation among populations (67% in women and 86% in men). This large contribution to CAD incidence variation among populations might be a result of colinearity with several other common genetic and environmental factors. These results are not consistent with the cardiovascular Mediterranean paradox for genetics and support a CAD genetic architecture mainly based on combinations of common genetic polymorphisms. Population genetic risk scores is a promising approach in public health interventions to develop lifestyle programs and prevent intermediate risk factors in certain subpopulations with specific genetic predisposition.

## Introduction

The development of coronary artery diseases (CAD) is the result of complex interactions between numerous environmental factors and genetic variants at many loci. Consequently, understanding CAD needs a multidisciplinary research effort.

Initially, epidemiologic research was largely based on cohort studies and clinical trials identifying and quantifying the relative importance of risk factors. As the World Health Organization (WHO) stated, the main identified risks for heart disease are behavioral factors: unhealthy diet, physical inactivity, tobacco use or harmful use of alcohol are present in about 80% of coronary events [1]. Different consortia had contributed to the development of estimation risk charts based on traditional risk factors (TRF), such as the Framingham Risk Score [2], the Reynolds Risk Score [3], the Prospective Cardiovascular Munster Heart Study (PROCAM) [4] and the Systematic Coronary Risk Evaluation (SCORE) system [5]. The prediction ability of these risk estimates is moderate-good [6]. Nevertheless, it has been estimated that nearly 15–20% of CAD patients are misclassified as “low risk” by TRF-based charts [7].

Genetics provided a plausible explanation for disease outcome in people without previous symptoms, and to the observed symptomatic variability in people exposed to similar behavioral risk factors. At the time that genetic disease architecture was partially unveiled, the idea of improving cardiovascular risk prediction was targeted. However, lack of replication, modest genetic risks and the small proportion of heritability explained by genome-wide association (GWA) studies have prevented the improvement of genetic CAD prediction [8], [9]. Even polygenetic risk scores, proposed as a way of improving already existing estimation risk charts, have not been completely satisfactory in different epidemiologic samples [10]–[16]. All the approaches mentioned above used individuals as the units of analysis.

From another perspective, in which general populations were the units of analysis, ecological/epidemiological (from now on: eco-epidemiologic) studies have assessed the disease population burden through the geographic distribution of CAD incidence and risk parameters. In this field, an important contribution has been made by the international WHO MONICA Project [17] which surveyed 38 populations from 21 countries. As far as CAD mortality was concerned, early cross-sectional studies reported an existing four-fold gradient across populations in the European continent, from the South (Spain and France) with the lowest CAD mortality towards the North (Finland and UK) [18]–[20]. Several studies have attempted to correlate this observed CAD incidence variation with the distribution of both traditional and genetic risk factors. It was assessed that classical risk factors contribute to 30–40% of CAD population incidence. Furthermore, an ecological fallacy was described when populations with remarkable differences in coronary mortality had similar classical risk factor levels, especially animal fat intake [21]–[23], leading to the idea of a French, southern European or Mediterranean paradox. However, a more recent study pointed towards wine consumption as an alternative explanation for this phenomenon [24]. The lack of strong correlations between CAD incidence and traditional risk factors suggests that genetic variation could be behind the interpopulation gradient of coronary mortality.

Some researchers have analyzed the geographic distribution of genetic risk variants to predict variation in both TRF and CAD mortality. So far, these studies have demonstrated that only the APOE*E4 risk allele is clearly correlated with CAD incidence among MONICA populations in the European continent. The lack of correlation for the vast majority of tested genetic markers led to extending the Mediterranean paradox to genetics [25], [26].

This study proposes an alternative approach to estimating the population genetic CAD burden using genetic risk scores (GRS). GRS appear to be a more realistic tool because they summarize the potential multiple risk genetic influences into a single quantitative parameter and do not depend on single genetic variants. As far as we know, no previous epidemiology studies have considered GRS as ecological risk predictors of CAD incidence. In order to describe geographic patterns of genetic risk variation, this study maps the population mean GRS using the geostatistical method known as kriging. Kriging is a geostatistic method for interpolating the spatial distribution of a variable by means of linear regression. Contour maps depicting interpolated spatial distribution patterns have previously been used to represent biologic anthropological data [27].

In order to explore these population approaches, we focused on a key piece of the CAD jigsaw: the role of nitric oxide (NO) in regulation of vascular tone homeostasis, tissue perfusion, and platelet aggregation [28], [29]. Three nitric oxide synthases (*NOS*) are responsible for NO availability: *endothelial NOS* (*eNOS* or *NOS3*), *neuronal NOS* (*nNOS* or *NOS1*) and *inducible NOS* (*iNOS* or *NOS2A*). *NOS3* and *NOS1* are constitutively expressed mainly in vessel endothelium and neuronal tissue, respectively [28]. Both are acutely regulated through reversible calcium-calmodulin binding. Conversely, *NOS2A* is activated through inflammatory signals in critical situations, mainly in the vessel endothelium and macrophages. However, induction of high-output *NOS2A* may lead to direct oxide cell toxicity or interfere with the beneficial activities of constitutive *NOS* isoforms [28]. Besides *NOS*, sONE is an antisense mRNA derived from a *NOS3AS* or *ATG9B* transcript unit on the complementary DNA strand from which the *NOS3* mRNA is transcribed. *ATG9B* and *NOS3* genes are oriented in a tail-to-tail configuration, and the mRNAs encoding sONE and NOS3 overlap for 662 nucleotides. There is evidence supporting a role for *ATG9B* in the post-transcriptional regulation of *NOS3* expression [29]. According to the Human Genome Epidemiology (HuGE) Navigator browser (www.hugenavigator.net), the *NOS3* gene is the second most reported gene for CAD, with 134 related papers, and the fourth most reported for myocardial infarction, with 74 reports (February, 2014). Variation in *NOS3* has also been tested for hypertension and diabetes. However, large meta-analysis on *NOS3* gene polymorphisms reported inconsistent results for CAD [30]–[33] and hypertension [34], [35] showing an excess of positive results associated with small sized studies and Asian populations. *NOS1* and *NOS2A* genes have been associated with CAD, hypertension, inflammation and diabetes [36]–[39], but also with a broader spectrum of diseases. All the above mentioned association studies only considered a few polymorphisms per gene region, and no one surveyed these chromosomal regions with a dense genetic coverage.

In this context, the present work had three main objectives. The first objective was to assess the prediction ability of GRS computed from *NOS* risk variants detected by association analyses among CAD patients and control samples. The second objective was to estimate, for the first time, the population *NOS* CAD burden computing GRS in general population samples, and to describe geographic patterns of GRS across Europe and the Mediterranean area. The third objective was to assess whether the population GRS are able to predict ecological risk. With this aim, population GRS were correlated with population distribution of CAD incidence and other traditional risk factors reported by the MONICA Project.

## Materials and Methods

### Ethics statement

The study has been specifically approved by the Ethical Committee of the University of Barcelona (Institutional Review Board: IRB00003099) and all the participants provided a written informed consent.

### Association and prediction analyses sample description

DNA samples of 324 myocardial infarction (MI) patients and 366 controls from the general Spanish population, obtained from the Spanish National DNA Bank (NDB) (www.bancoadn.org), were genotyped in this study. This sample will be referred to from now on as NDB cardiovascular (NDBC) sample. Additionally, genotype data from four European matched case-control samples from the Myocardial Infarction Generation (MIGen) Consortium [FINRISK (Finland), MDCS (Sweden), ATVB (Italy) and Regicor (Spain)] were obtained through the database of Genotypes and Phenotypes (dbGAP; www.ncbi.nlm.nih.gov/gap) [40]. In summary, a total of 2575 MI cases and 2617 controls were used in this stage. Extensive details on the clinical characteristics of these samples have been previously described (www.bancoadn.org/en/introNCa.htm) [40]. Briefly, fatal and nonfatal MI were reported or diagnosed by general practitioners based on autopsy reports, electrocardiographic data, cardiac biomarkers, and additional clinical information.

### Eco-epidemiologic analyses sample description

A total of 34 populations (n = 1663 individuals) from Europe, North Africa, and the Middle East were analyzed (see Figure S1). Thirty populations (n = 1298) corresponded to healthy unrelated individuals of both sexes that were genotyped in the present study and whose four grandparents had been born in the same geographical region. Additionally, genetic data from four other European samples from the 1000 Genomes Project [41] were included in the analyses.

### Polymorphisms and genotyping

The NDBC sample (324 cases and 366 controls) and 1298 individuals from the 30 general populations were genotyped for 78 single nucleotide polymorphisms (SNP) using a GoldenGate Genotyping Assay (Illumina Inc., San Diego, CA). This SNP set was selected as being representative of the common variation in the three genomic regions of the *NOS* genes, with an average coverage of 1 SNP every 5 kb with a minor allele frequency higher than 0.05 (MAF>0.05) in the CEU population as reported in the HapMap project (www.hapmap.org). Out of the 78 determined SNPs, 13 were located in chromosome 7 spanning 41.4 kb in the *NOS3* and *ATG9B* genes region; 43 SNPs in chromosome 12 that include the *NOS1* gene along 177.4 kb, and 22 SNPs in chromosome 17 covering 92.2 kb in the *NOS2A* gene region. SNP details are shown in Table S1 in File S1.

Genotype data for the MIGen samples were generated in the corresponding original project using the Affymetrix 6.0 GeneChip [40].

### Quality control and imputation

Genotyping rate, allele frequencies, and deviations from the Hardy-Weinberg equilibrium (HWE) were calculated using PLINK software [42]. SNPs with a genotyping rate lower than 0.75 or not polymorphic in any sample were removed from the analysis. Individuals with a genotyping rate lower than 0.75 or not genetically homogeneous compared with individuals of the same population group were also removed for the analysis. Missing genotypes were inferred using MACH 1.0 software [43] taking as reference the rest of the genotypes ascertained in the same population. Linkage disequilibrium was calculated and visualized using Haploview software [44].

Datasets from the MIGen study already included 27 out of the 78 SNPs in the *NOS* regions. In order to have the same genetic information, SNPs not directly genotyped in the MIGen samples were imputed using two different imputation softwares, MACH 1.0 [43] and IMPUTE2 [45]. In both imputations the computational effort was controlled performing 200 algorithm iterations when phasing and imputing data sets, and considering 300 haplotypes to use as templates when phasing observed genotypes. This imputation effort is four times higher than the standard effort recommended by software developers. Phased chromosomes from the most similar 1000 Genomes Project samples were used as reference panels: the FIN sample for the FINRISK case-controls, the TSI sample for the ATVB and Regicor case-controls, and the CEU sample for the MDCS case-controls.

As a control approach to validating the genotyping strategy of this study (SNPs selected as representative of *NOS* regions common variation), in our population sample from Central Italy (CIT) we imputed all the variation described in TSI sample from the 1000 Genomes Project in the studied three chromosomal regions. And then we checked the imputation quality indices regarding allele frequency thresholds.

### Association and prediction analysis

A two-step analysis of association and prediction was performed with the *PredictABEL* R package [46]. These analyses were performed in duplicate, in the MACH imputed data set and in the IMPUTE2 imputed data set. In the first step, associations were tested by logistic regression analysis in the three case-control samples with the largest sample size: FINRISK from northern Europe, and ATVB and Regicor from southern Europe. The other two case-control samples (MDCS and NDBC) were kept as cross-validating samples for the posterior prediction step. In the association analyses, only SNPs with a LD measure (r^2^) lower than 0.8 between pairs and imputation quality indexes (r^2^ for MACH 1.0 and i for IMPUTE2) higher than 0.6 in all three used case-control samples and the two imputation methods were included. Estimates of beta coefficients for each SNP were obtained using multivariate logistic regression analyses and adjusted for age, gender and the remaining genetic variables. In order to get a single robust estimate of the level of association for each genetic marker, a meta-analysis of the three previous association analyses (n = 4318) was conducted with the METAL software [47].

In the second step, NOS genetic risk scores for MI were computed in all five case-control samples. Risk scores were constructed using allele dosages of low P value (p<0.1) risk alleles identified in both meta-analysis from MACH and IMPUTE2 data sets. Thus, homozygotes for the reference allele were coded as 0 and homozygotes for the risk allele as 2. The risk SNPs were pruned by LD (r^2^) lower than 0.2 in order to obtain a set of unequivocally independent SNPs to calculate the risk scores. This LD pruning was performed by Tagger [48], implemented in Haploview [44], preferentially picking the SNPs with the lowest P value. As an approach to checking for the epidemiological relevance of the estimated risk scores, predictive models were constructed based only on these *NOS* risk scores in all five case-control samples. These models were performed to assess the fraction of interindividual variance of the MI affection status explained by *NOS* risk score through Nagelkerke's R^2^. Moreover, discrimination accuracy of the *NOS* risk score between patients and healthy controls was estimated as the area under the receiver operating characteristic (ROC) analysis curve (AUC) index.

### Eco-epidemiologic analysis

*NOS* genetic risk scores for MI were computed in the general population samples as previously described. *NOS* risk scores were tested for normality in each population sample using the *nortes*t R package. Spatial distribution of mean risk score across populations was mapped using the geostatistical method known as kriging from the ArcGIS software (ESRI, Redlands, CA, USA). Since anisotropy was not detected in the semivariogram, we used the ordinary spherical interpolation kriging method [27]. The covariation of the observed spatial distribution with geography was assessed by Moran's I and Geary's C randomization tests for spatial autocorrelation [49] using *ade4* R package. Also, the spatial structure of mean risk scores was assessed using correlograms, which estimate autocorrelation coefficients for different spatial relationships, with the PASSaGE software [50]. Population pair relationships were classified in different classes representing increasingly larger distances. Autocorrelation coefficients were then calculated for each distance class and plotted against distance [51]. Data related to coronary event rates and prevalence of traditional risk factors in middle-aged individuals were compiled from the MONICA Project [52] for the 11 European populations genetically tested here. Among the genotyped populations in this work, four of them (POL, NFR, SFR, and CAT) had a MONICA counterpart and seven additional populations (ORK, GBR, CEU, FIN, TSI, CIT and NBH) had a MONICA population within a 200-km radius or from the same country. The CEU sample was considered counterpart of the MONICA German-Bremen population according to Lao et al. [53]. Average annual coronary event rates over 5 years and average levels of systolic blood pressure (SBP), total cholesterol (TCH), body-mass index (BMI), and daily smoking rate (SMK) by gender were obtained from Kuulasmaa et al. [52].

Spearman's correlation and univariate linear regression analyses were performed to estimate the contribution of traditional risk factors to population variation in coronary event rates using the *stats* R package. Since *NOS* genes are involved in blood pressure homeostasis, *NOS* genetic parameters (population mean risk scores and allele frequencies) were also correlated and regressed with population variation in coronary event rate and systolic blood pressure levels. Moreover, since geography could underlie the distribution pattern of several environmental and genetic risk factors, latitude and longitude were also tested for correlation and regression with coronary event rates. Finally, multivariate regression analyses were performed with factors that were significant in univariate analyses in order to estimate the contribution of genetic risk factors beyond geography.

## Results

### Genotyping, quality control, and imputation

Genotyping rates and status for the 78 SNPs initially tested in our samples are shown in Table S1 in File S1. Genotyping rates ranged from 81.01 to 91.21%. Ten SNPs were not successfully genotyped, and three SNPs were not polymorphic in the tested populations. These 13 SNPs were removed from the study. As for the data coming from the international project, genotyping status and imputation quality indexes are presented in Table S1 in File S1. Four SNPs had an imputation quality lower than 0.6 in at least one case-control sample used in the association analyses. Hence, 61 SNPs were considered consistent for analytical epidemiologic analyses.

After quality control, a total of 5096 samples for the epidemiologic survey and 1298 for the population analysis were included. The largest case-control sample was ATVB with a total of 3352 individuals, and the smallest samples were FINRISK and MDCS with 339 and 184 individuals (Table S2 in File S1). Among the general populations, sample sizes ranged from 32 to 50 individuals except the populations from the 1000 Genomes project (n = 85–98) as can be seen in Table S3 in File S1.

Minor allele frequencies (MAF) can be found in Table S4 and Table S5 in File S1 for case-control samples imputed with MACH and IMPUTE2 respectively, and in Table S6 in File S1 for population samples. None of the SNP showed significant departures from Hardy-Weinberg equilibrium expectations in any case-control or population sample (data not shown). Concerning linkage disequilibrium patterns, our data indicate that the three *NOS* genes are not regions of high LD. For instance, the LD pattern of the three *NOS* regions in the CEU sample can be visualized in Figure S2 for *NOS3* gene, Figure S3 for *NOS1* gene and Figure S4 for *NOS2A* gene. LD values were similar in the different case-control samples used in this study. After applying the LD pruning criteria for association analysis, 38 SNPs with low LD (r^2^<0.8) were considered.

Assessing the validity of our genotyping strategy, 71% of the common variants (MAF>0.1) present in the 1000 Genomes TSI sample were imputed with high accuracy (MACH r^2^>0.75) in our population samples from Central Italy, CIT (Table S7 in File S1). Hence, this result indicates that our genotyping strategy (1SNP each 5kb) efficiently captures more than 70% of the common variation reported by the 1000 Genomes Project in the three genomic regions.

### Meta-analysis and interindividual prediction analysis

Five SNPs had low P value (p<0.1) in both MI meta-analysis from MACH and IMPUTE2 data sets, four in the *NOS3* and one in the *NOS1* gene regions (highlighted in Table S4 and Table S5 in File S1). After LD pruning criteria (r^2^<0.2) to select completely independent SNPs, four SNPs remained dropping one SNP from *NOS3*. Genetic effects of these four SNPs with their standard error were similar for meta-analyses from MACH and IMPUTE2 data sets (Table 1). Association parameters of all tested markers for both multivariate logistic regression analyses and for both meta-analysis are shown in Table S4 and Table S5 in File S1.

**Table 1:** Meta-analyses association parameters of risk variants with P value<0.1 in both analysis (from MACH and IMPUTE2 datasets) after being pruned by LD lower than 0.2.

ID reference	Gene	Alleles	Risk Allele	MACH Effect	SE	P value	IMPUTE2 Effect	SE	P value
rs3793342	NOS3	A/G	G	0.18	0.10	0.071	0.20	0.10	0.046
rs1799983	NOS3	T/G	G	0.44	0.16	0.006	0.53	0.18	0.004
rs3918188	NOS3	A/C	C	0.29	0.09	0.001	0.30	0.09	0.001
rs3782219	NOS1	T/C	C	0.15	0.09	0.088	0.18	0.09	0.055

SE: Standard Error.

The assessment of interindividual predictive ability was almost identical using MACH and IMPUTE2 imputations and showed limited power in differentiating between cases and controls (Table 2 for MACH data set, and Table S8 in File S1 for IMPUTE2 data set). The distribution of *NOS* risk scores was similar in cases and controls, showing almost overlapping distributions. Besides, *NOS* risk score explained less than 1% of interindividual variance in affection status according to the Nagelkerke's R^2^. *NOS* risk score was only a significant discriminating factor in the ATVB case-control sample. This was also reflected by the discrimination accuracy assessed through AUC index slightly higher than 0.5 in this sample.

**Table 2:** *NOS* genetic risk score (GRS) distribution for cases and controls and discrimination accuracy for MACH imputed dataset.

Case-control sample	Mean risk score ± SD [min - max]		Nagelkerke's R^2^	AUC [95%CI]
	cases	controls		
FINRISK (Findland)	6.11±0.80 [4.00–7.97]	6.05±0.85 [3.96–7.98]	<0.01	0.517 [0.455–0.578]
ATVB (Italy)	5.88±0.68 [3.17–7.81]	5.81±0.70 [3.60–7.83]	<0.01	0.527 [0.508–0.547]
Regicor (Spain)	5.79±0.70 [3.88–7.46]	5.88±0.71 [3.92–7.87]	<0.01	0.470 [0.425–0.515]
MDCS (Sweden)	5.84±0.72 [4.01–7.67]	5.96±0.64 [4.47–7.75]	0.01	0.448 [0.364–0.532]
NDBC (Spain)	5.79±0.84 [4.00–8.00]	5.85±0.88 [3.00–8.00]	<0.01	0.469 [0.426–0.513]

SD: Standard Deviation; Nagelkerke's R^2^: explained interindividual variance of MI by *NOS* risk score predictive model; AUC: Area Under the receiver operating characteristic (ROC) curve; CI: Confidence Interval.

### Population distribution of *NOS* risk scores

**Figure 1:**
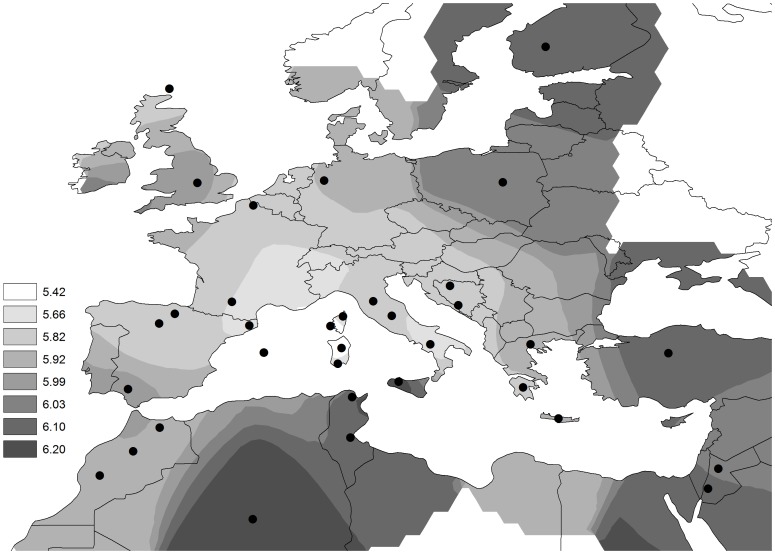
Contour map of *NOS* risk score in the European and Mediterranean samples.

The geographical distribution of mean *NOS* risk scores across European and Mediterranean populations is represented in a smoothed spherical contour map in Figure 1. Population values are shown in Table S9 in File S1. The lowest score values corresponded to southwestern European populations, specifically to the islands of Corsica and Sardinia (<5.5 risk alleles), North-East Spain (5.65) and South Italy (5.69). Variation in the map fitted a global pattern of concentric distribution departing from a center of low risk score values in the North-West of the Mediterranean Basin and gradually increasing according to geographical distances. This concentrical pattern significantly covariated with geography as reflected by the spatial autocorrelation analysis (p = 0.024). At European continental level, the pattern is consistent with a gradual increase towards North and North-East, with highest values in Great Britain (6.04), Poland (6.07) and Finland (6.14). A similar cline is also observed in the northern shore of the Mediterranean, with increasing values from Spain to Turkey (6.14) and Middle-Eastern populations, GJD (6.02) and BJD (6.22). This clinal pattern in the European continent and the Middle East was statistically assessed plotting Moran's I and Geary's C autocorrelation coefficients by distance between population pairs (Figure S5). Six distance classes of population pairs with an average of 30 observations per class were obtained. Autocorrelation coefficients for population pairs at short distances denoted significant positive autocorrelations while for population pairs at long distances Moran's I and Geary's C coefficients detected negative autocorrelations. Concerning the distribution between the northern and southern Mediterranean shores, a gradual variation can be observed in the westernmost part (Spain and Morocco), but the pattern is sharper in the central part of the region (i.e. Tunisia and Italy).

### Eco-epidemiology of *NOS* gene variation and cardiovascular events

The eleven populations with both *NOS* genotype data and MONICA information are shown in Table S10 in File S1. From the MONICA parameters, only daily smoking rate appeared as slightly correlated with coronary event rates in women (rho = 0.57; p = 0.064) and explained 39% of the population variance of coronary event rates (p = 0.022).

**Figure 2:**
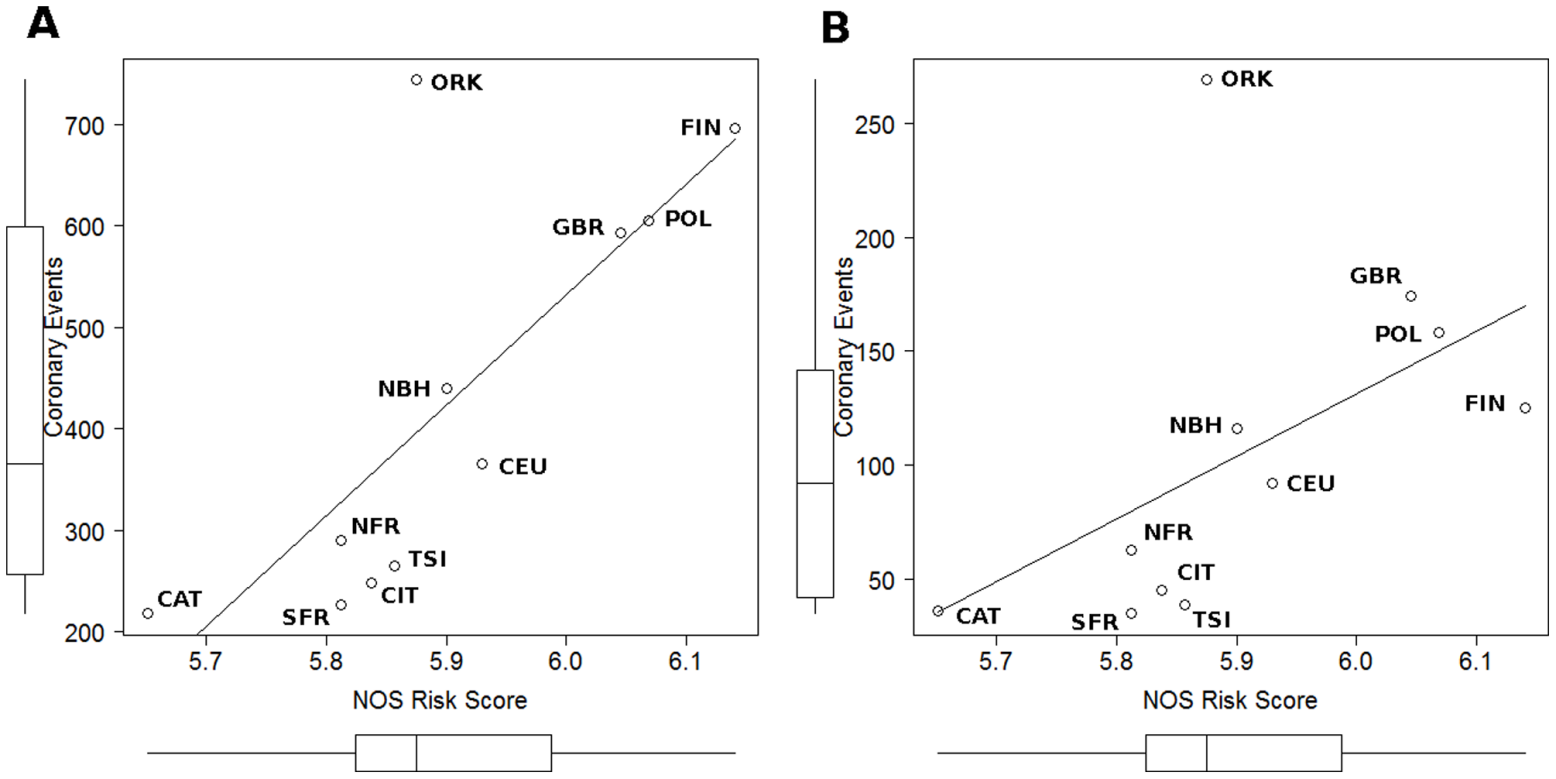
Correlation plots between average *NOS* risk scores and coronary events in men (A) and women (B). Coronary events: rates per 100,000 people from the MONICA project.

Mean *NOS* risk score values in the eleven populations considered were positively correlated with coronary event rates in men (rho = 0.82; p<0.01) (Figure 2A) and women (rho = 0.76; p<0.01) (Figure 2B). In these figures, the ORK sample appeared as substantially different from the others. In the regression analysis, variation in mean risk scores explained 53% of interpopulation variance in coronary event rates in men and 19% in women (Table 3), and the ORK sample was confirmed as an outlier sample (Bonferroni p<0.01 for both men and women). The ORK sample and its MONICA counterpart were excluded from the regression analyses. The outlier character of the Orkney Islands sample was probably due to an island genetic drift phenomenon when only a few markers are analyzed. Following this trend, any MONICA parameter was correlated with coronary events in the remaining ten MONICA samples, and the *NOS* risk score of the ten continental samples significantly explained 86% of coronary events in men and 67% in women (Table 3). Regarding SBP, 35% for men and 27% for women of the population variance was accounted for by the *NOS* risk score (Table 3). Individually, frequency distributions of 11 SNPs were associated with population coronary event rates or systolic blood pressure levels in men or women as estimated by correlation and univariate regression analyses (Table 3). Eight SNPs were correlated with coronary events in both men and women, explaining a remarkable proportion of the coronary rates variance: 34–67% in men and 36–52% in women. Out of these eight SNPs, 5 belonged to the shortest (42kb) region examined comprising the *NOS3* and *ATG9B* genes. This region was initially tested by nine SNPs, indicating that most of the tested genetic variation of this region had a similar geographic distribution pattern to coronary event rates. Among these SNPs correlating with CAD incidence, only the G risk allele of the rs1799983 was included in the risk score. Moreover, the same allele was the only one to be positively correlated with SBP in both men and women, explaining similar proportions of interpopulation variation, 40% in men (p<0.05) and 51% in women (p<0.01) (Table 3).

**Table 3:** Spearman's Rho and % of explained interpopulation variance (adjusted R^2^) of coronary event rates and SBP by gender of significantly correlated minor allele frequency (MAF) variants and population *NOS* risk scores.

			Correlation and univariate linear regression analysis in men				Correlation and univariate linear regression analysis in women			
			Coronary event rate		SBP		Coronary event rate		SBP	
			rho	R^2^	rho	R^2^	rho	R^2^	rho	R^2^
**NOS GRS**			0.82**	0.53**	ns	0.36*	0.76**	0.19'	0.52'	0.28'
**Continental NOS GRS**			0.93***	0.86***	ns	0.35*	0.85**	0.67**	ns	0.27'
**ID reference**	**Gene region**	**MAF Allele**								
rs11771443	NOS3	T	0.68*	ns	ns	ns	ns	ns	ns	ns
rs1799983	NOS3	T	−0.85**	0.61**	−0.80**	0.40*	−0.79**	0.45*	−0.86**	0.51**
rs753482	NOS3	G	−0.82**	0.65**	ns	ns	−0.71*	0.52*	ns	ns
rs7830	ATG9B	T	0.84**	0.56**	ns	ns	0.74*	0.49*	ns	ns
rs2373929	ATG9B	T	0.78**	0.49*	ns	ns	0.63*	ns	ns	ns
rs13307588	ATG9B	A	−0.83**	0.48*	ns	ns	−0.72*	0.38*	ns	ns
rs10774907	NOS1	A	0.87**	0.67**	ns	ns	0.81**	0.64**	ns	ns
rs2271986	NOS1	T	0.65*	ns	ns	ns	0.71*	ns	ns	ns
rs1889024	NOS2A	G	ns	0.33*	ns	ns	0.63*	ns	ns	ns
rs953527	NOS2A	A	ns	ns	ns	ns	ns	ns	0.65*	ns
rs8072199	NOS2A	T	0.75*	0.34*	ns	ns	0.65*	0.36*	ns	ns

‘: P value<0.1;

*: P value<0.05;

**: P value<0.01;

***.P value<0.001. ns: non-significant.

Since north-to-south and east-to-west geographic patterns of variation in genetic and environmental risk factors could underlie the observed associations between polymorphisms in *NOS* regions and coronary events, we then assessed the influence of geography in the distribution of coronary events. Latitude was strongly correlated in men (rho = 0.87; p<0.01) and women (rho = 0.75; p<0.05) explaining a high proportion of population incidence (66% in men and 38% in women).

Grouping the different parameters that were independently correlated with coronary events rates, a multivariate analysis was performed including the *NOS* risk score and the latitude in both male and female models. The result showed latitude was no longer significant, and the proportions of population coronary incidence for both men and women accounted for by the multivariate models were not higher compared with the *NOS* risk score univariate models. For men, the multivariate model explained 85% of population coronary incidence (p<0.001; NOS risk score p = 0.01, latitude p = 0.45). And for women, it explained 62% of the population coronary events rate (p = 0.01; NOS risk score p = 0.04, latitude  = 0.82).

## Discussion

This study analyzes the role of molecular variation from *NOS* genes in cardiovascular patients and assesses the population distribution of genetic risk scores as an ecological predictor of the CAD burden across the European and Mediterranean landscape for the first time. The *NOS* GRS included the 4 genetically independent SNPs with lower P value associated with MI in a meta-analysis of three European case-control studies. Since *NOS* genes regulate the physiological availability of NO, this GRS constitutes a polygenic approach to the potential contribution of NO to CAD. The interindividual contribution of the GRS was lower than 1%. However, from an ecological perspective, GRS values across Europe were positively correlated with the incidence of coronary events explaining 65–85% of interpopulation variation of CAD incidence. These contrasting contributions and the usefulness of GRSs as ecological predictors are discussed below.

### Interindividual (intrapopulation) and interpopulation contribution to CAD of *NOS* GRS

In the context of case-control studies, the *NOS* GRS was only a significant MI risk factor in the sample with the largest size (ATVB) but not in the other studies. Also, the AUC indicated that the predictive value of the GRS was null or very limited (AUC of 0.527).

The weak effects of our GRS performed in terms of both association and prediction are in complete accordance with previous reports in the literature. The proportion of variance explained by the relatively large number of loci associated with CAD is lower than 1% [54]. In addition, the improvement in risk prediction provided by genetic markers appeared to be null or insufficient [55], even with the strongest and most replicated CAD risk factor identified in the 9p21.3 locus [7], [56]. When we move from single genetic risk variants to a genomic profile, the combined effect of dozens of risk variants generally explains only a small proportion of disease variance [56] and shows a limited predictive ability (AUC of 0.55–0.62) [6], [55]. For instance, in the MIGen Consortium, the effect of the nine top-associated loci explained 2.8% of phenotype variance [40]. Even a more comprehensive genetic risk score of 101 SNPs associated with MI and other cardiovascular risk factors explained less than 5% of interindividual variance [57]. In spite of the low genetic contribution of genomic profiles, the genetic basis of CAD is strong as reflected in family aggregation data (40% for women and 60% for men) [6], [7], [58]. The proportion of heritability that remains unaccounted for (referred to as “missing heritability” elsewhere) would be explained by common genetic variants (MAF >0.05) having very small effects and rare variants with a larger contribution to the complex phenotype [9], [58]–[60].

The geographic distribution of GRS presented an interesting variation pattern across European and Mediterranean populations. This distribution showed a concentric pattern from a center of lower risk scores in North-Western Mediterranean, specially the Islands of Corsica and Sardinia (Figure 1). The gradual increase towards North (UK) and North-East Europe (Poland and Finland) through the scarcely sampled area of North-Central Europe does not seem unreasonable given the general trends across the European continent and does not suggest any major problems with spurious interpolation. This European south-to-north cline in population GRS explained a large proportion of variance in coronary incidence across 10 MONICA populations, 67% in women and 86% in men (Figure 2). This large contribution contrasts with the intrapopulation (i.e. interindividual) contribution of the GRS (<1%). Looking at single genetic markers to understand this phenomenon, we have identified some genetic variants, mainly in the *NOS3/ATG9B* region, with frequencies correlating with CAD incidence. According to these correlations all these variants would explain a similar proportion of variance in CAD incidence (35–65%), but lower than GRS (Table 3). Out of this group of correlating variants only rs1799983 was included in the GRS. The other genetic variants correlating with CAD incidence were not associated with CAD phenotype. In the literature, empirical data on the ecological applications of GRSs are lacking, but some studies have been done using single markers. Previous studies on the correlation between risk allele frequencies and CAD incidence across MONICA populations did not find conclusive results [25], [26]. One of these studies [26] extended the Mediterranean paradox discussion into genetics due to the observed negative correlations between some genetic risk factors and CAD events. These authors concluded that the observed north-to-south cline in the frequency of some genetic risk variants was most probably the result of spatial distribution of the whole genome variation present in the European continent, which has been mainly shaped by the history of populations [61]–[63]. In this context, the variance explained by the markers correlating with CAD incidence in this study cannot be considered a specific effect of each variant, but rather the combined effect of many risk variants showing the same distribution. Thus, the apparent high effect (46% in women and 61% in men) of the risk SNP rs1799983 is likely to be a colinearity effect with other risk genetic factors in the regression analysis. In the same way, the estimated contribution of GRS (67% in women and 86% in men) would be the consequence of the joint effect of risk variants from the common frequency spectrum, with similar population patterns as the 4 SNPs included in the GRS. In other words, the contrasting contributions from interindividual variance (<1%) to interpopulation variance (65–85%) suggests that this GRS population approach most probably suffers from colinearity with other genetic as well as environmental risk factors. These results would support that a high proportion of population CAD incidence is determined by common genetic variant distributions because classical risk factors contribute in 30–40% of CAD population incidence, and because rare variants are basically population-specific and are not distributed in population gradients [41], [60].

The important genetic contribution to CAD incidence variation suggests some considerations about the role of genetic factors on the individual risk to CAD. High incidence of CAD in a population would be determined by high frequency of genetic risk combinations and, hence, a high proportion of individuals carrying these genetic risk combinations. In this ecological approach, the GRS is capturing the contribution to CAD incidence of the myriad of genetic risk variants with similar geographic distribution. Thus, only the joint inclusion of this myriad of genetic variants would explain a considerable proportion of the genetic contribution to CAD outcome at the individual level. In accordance with these results, previous studies have dealt with the number and the effect sizes of the genetic variants involved in the genetic architecture of CAD. These simulation models predicted that the number of genetic variants needed to explain the estimated heritability, under a purely additive model, would range from few hundred low frequency variants with large effect to several hundred or few thousand for common variants with small effect [6], [64], [65].

In addition to the "Finland-to-Spain" axis, the variation in GRS is also clinal between the West and East in the northern Mediterranean shore. Although the correlation with CAD incidence is not easy to demonstrate due to a sizeable lack of epidemiological data in these populations, some partial data [66] point in this direction. This constitutes an additional insight into our working hypothesis on the correlation between CAD incidence and GRS in Europe. So, the population distribution of both our GRS and cardiovascular incidence are deeply influenced by similar demographic processes that have modulated genetic variation in current human groups. Available data in North Africa [67] are too scarce to extend any conclusion to the southern shore of the Mediterranean.

### Usefulness of population GRS approach

In the European continent, environmental factors as well as genetic variation seem to be structured in south-to-north clines that can be correlated with observed CAD incidence as reflected by latitude in our study. In previous data such as the original MONICA project, modifiable risk factors explained only 30% of coronary incidence variance [24]. And in this study, multivariate analyses stressed the importance of GRS to explain the distribution of CAD incidence, especially in men. These results reflect that most variance in CAD incidence among populations is accounted for by genetic background. A priori, this would contrast with the fact that at the interindividual level (within a population) environmental and behavioral factors are involved in 80% of all cardiovascular events. However, they are two complementary sides of the same phenomenon that explain different features of the disease: individual outcome and population incidence. Whereas within a population environmental factors explain a large proportion of individual events, the total amount of coronary events in populations under similar environmental pressures would depend on their genetic predisposition. The potential incidence of CAD in a population would be mainly determined by its genetic risk background but it would be triggered by behavioral and life style factors.

Our results highlight the usefulness of GRSs as population estimates of the genetic burden of disease or as ecological predictors. It has been stated that the assessment of disease risk and its temporal trends is of critical importance to predict incidences of CAD [68]. An ideal GRS would include all CAD risk variants in the genome. Nevertheless, an exploratory strategy could include the design of different GRSs for different pathophysiological processes related to CAD, such as endothelial dysfunction, accelerated atherosclerosis or thrombosis, each one having its own genetic basis [68]. These GRSs could provide a solid basis for developing lifestyle intervention programs to prevent intermediate risk factors (e.g. obesity, high levels of blood pressure, glucose and lipids) in population subgroups before environmental factors trigger a potentially high genetic predisposition for the disease.

Another particular advantage of GRS is that they can be constructed from samples of a few hundreds of individuals per population. High potential benefits, no interventional harms and low cost make this approach very promising for future public health studies.

### Limitations

The analyses of this study have some limitations that can be commented on. A first aspect refers to the genotyping strategy; with the aim of capturing the maximum common genetic variation in *NOS* genes we genotyped 1 SNP every 5 kb in our samples (65 SNPs in total). The accuracy of this strategy was assessed by imputing all variants present in the TSI 1000 Genomes population sample in our general population sample from Lazio, Italy (genotyped with our set of 65 SNPs). From the result, we concluded that this genotyping strategy was representative of >70% of common genetic variation. Therefore, a remaining 30% of common genetic variation of *NOS* genes is not well represented in these analyses. Secondly, in MIGen case-control samples a considerable proportion of genotypes (∼60%) were imputed, and, even though imputation quality controls were performed, this fact could have affected the association and meta-analysis results. In any case, this fact does not invalidate the GRS population approach because the distribution of a robust (large number of polymorphisms) GRS does not depend on the distribution of single polymorphisms. Thirdly, the GRS in this study correspond only to a small piece of the genetic basis of cardiovascular diseases jigsaw. So, this initial study should be further developed beyond polymorphisms in *NOS* genes. Finally, the size of the general population samples was robust enough to check the frequency distributions of polymorphisms. However, future studies should include larger sample sizes if less common (<5%) polymorphisms are to be included.

## Conclusions

This study of cardiovascular *NOS*-GRS in European populations shows for the first time that GRSs are a powerful way of analyzing the distribution of genetic risk and a promising tool for ecological predictions of disease.

Although the contribution of GRS to CAD at the individual level was lower than 1%, GRS explained a large proportion of interpopulation differences in CAD incidence (65%–85%). This large contribution to CAD incidence across populations might be the result of colinearity with several other common genetic and environmental factors. From the GRS perspective, the so-called cardiovascular Mediterranean paradox would be no longer held and CAD genetic architecture would be mainly based on common genetic polymorphisms. The genetic risk score population approach seems very promising in future public health interventions to develop lifestyle programs and prevent intermediate risk factors in population subgroups with especially high genetic predisposition.

## Supporting Information

Figure S1 **Geographic population distribution of European and Mediterranean samples.** See Table 3 in File S1 for abbreviation codes.
(TIFF)

Figure S2 **Plot of linkage disequilibrium (r^2^) between tested genetic markers from** ***NOS3/ATG9B*** **region in CEU sample.**
(TIFF)

Figure S3 **Plot of linkage disequilibrium (r^2^) between tested genetic markers from** ***NOS1*** **region in CEU sample.**
(TIFF)

Figure S4 **Plot of linkage disequilibrium (r^2^) between tested genetic markers from** ***NOS2A*** **region in CEU sample.**
(TIFF)

Figure S5 **Correlograms of Moran**'**s I (A) and Geary**'**s C (B) autocorrelation coefficients for different distance classes of population pairs.** Distances in kilometers. Full circles mean significant coefficients.
(TIFF)

File S1 **Tables S1-S10.** Table S1. Genomic location of the genetic variants, genotyping and imputation details. Chromosome positions from Genome Reference Consortium human build 37 (GRCh37). GEN: Genotyped; IMP: Imputed. r^2^: MACH quality metric. i: IMPUTE2 quality metric. Table S2. Original project, sample size, gender ratio and age (mean, standard deviation and range) of case-control samples. Table S3. Geographic origin, population codification, sample size and geographic coordinates in decimal degrees for the population samples. Table S4. Case-control allele frequencies and association parameters of MAF variants from MACH imputated data. LIQ: Low imputation quality. LD: Excess linkage disequilibrium. SE: Standard Error. ‘: P value<0.1; *: P value<0.05; **: P value<0.01. Table S5. Case-control allele frequencies and association parameters of MAF variants from IMPUTE2 imputated data. LIQ: Low imputation quality. LD: Excess linkage disequilibrium. SE: Standard Error. ‘: P value<0.1; *: P value<0.05; **: P value<0.01. Table S6. Minor allele frequencies (MAF) for population samples. Table S7. Total number of polymorphisms presented in TSI 1000 Genomes Project sample (N polymorphisms) and rates of high accurately imputed (MACH r^2^ >0.75) polymorphisms in CIT population sample. Table S8. NOS genetic risk score (GRS) distribution for cases and controls and discrimination accuracy for IMPUTE2 imputed dataset. SD: Standard Deviation; Nagelkerke's R^2^: explained interindividual variance of MI by NOS risk score predictive model; AUC: Area Under the receiver operating characteristic (ROC) curve; CI: Confidence Interval. Table S9. Mean NOS risk score with standard deviation, minimum and maximum for population samples. Table S10. Present study/MONICA population pairs with coronary event rate and mean levels of traditional risk factors separated by gender. CER: Mean coronary event rates per 100,000 people over 5 years; SBP: Systolic blood pressure (mm Hg); TCH: Total cholesterol (mmol/L); BMI: Body-mass index (Kg/m^2^); SMK: Daily smoking rate (%).
(XLSX)
