# Supplementary figures and images for: Genetic Risk Score of NOS Gene Variants Associated with Myocardial Infarction Correlates with Coronary Incidence across Europe
Source: PLoS One. 2014 May 7;9(5):e96504. doi: 10.1371/journal.pone.0096504 (PMC4013019; doi:10.1371/journal.pone.0096504)

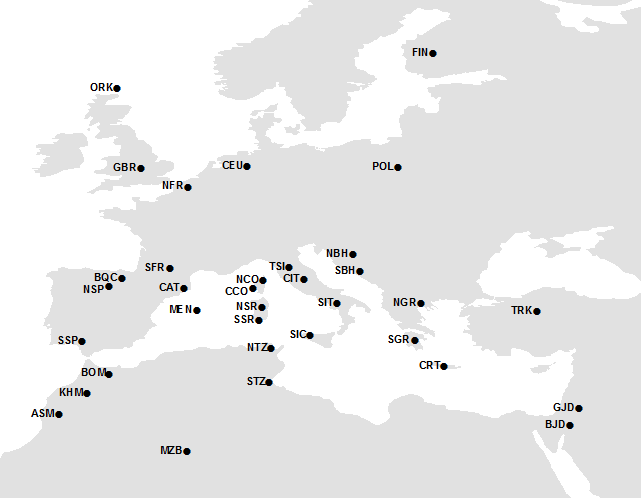

Supplement: Figure S1 — Geographic population distribution of European and Mediterranean samples. See Table 3 in File S1 for abbreviation codes. (TIFF) [file pone.0096504.s001.tiff]

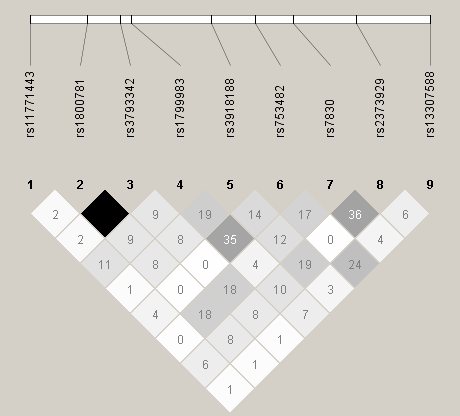

Supplement: Figure S2 — Plot of linkage disequilibrium (r2) between tested genetic markers from NOS3/ATG9B region in CEU sample. (TIFF) [file pone.0096504.s002.tiff]

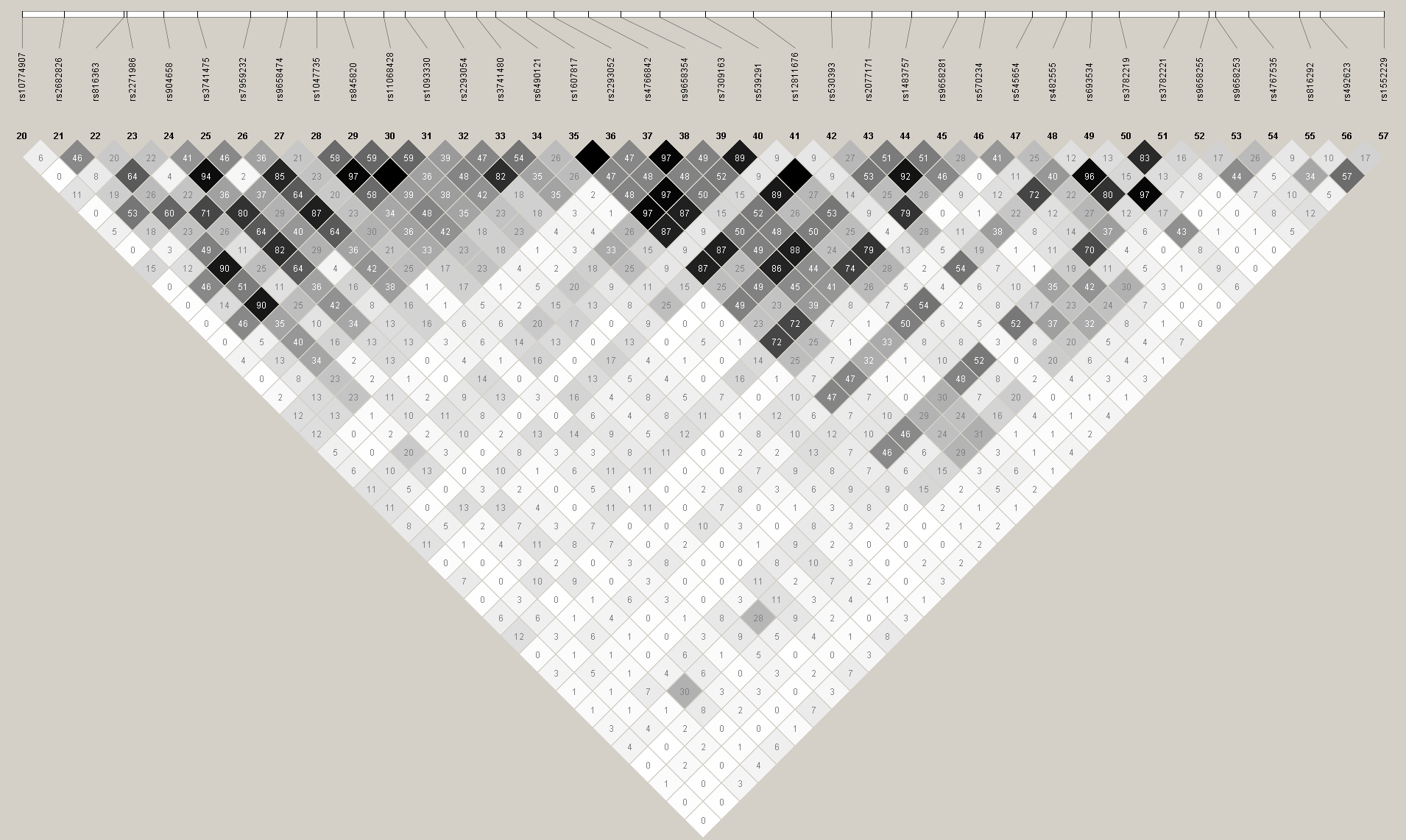

Supplement: Figure S3 — Plot of linkage disequilibrium (r2) between tested genetic markers from NOS1 region in CEU sample. (TIFF) [file pone.0096504.s003.tiff]

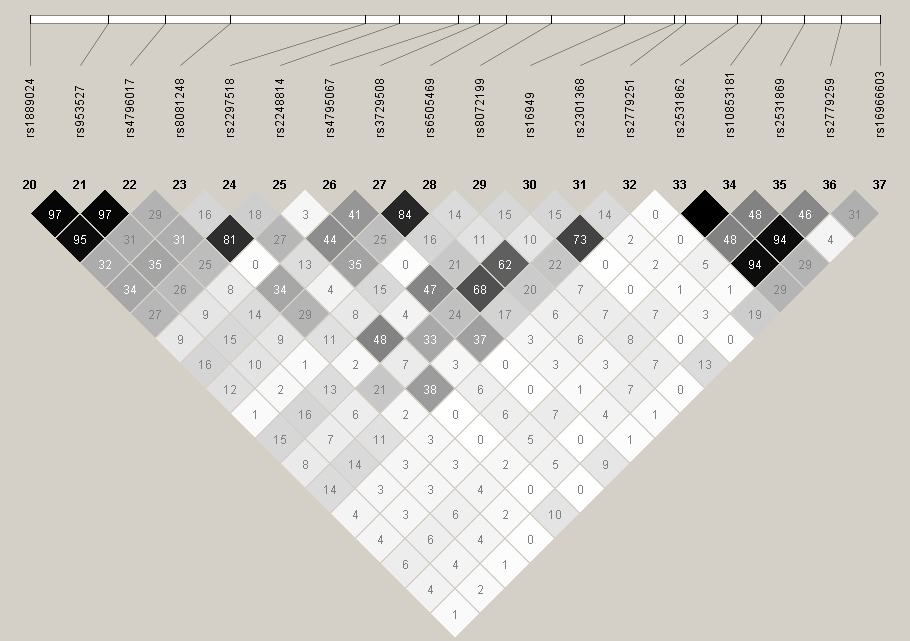

Supplement: Figure S4 — Plot of linkage disequilibrium (r2) between tested genetic markers from NOS2A region in CEU sample. (TIFF) [file pone.0096504.s004.tiff]

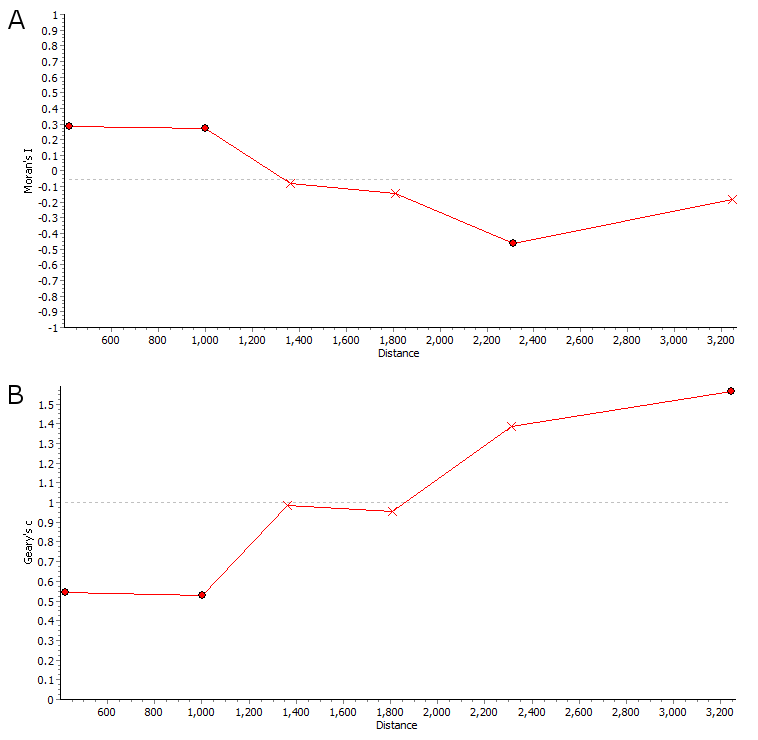

Supplement: Figure S5 — Correlograms of Moran's I (A) and Geary's C (B) autocorrelation coefficients for different distance classes of population pairs. Distances in kilometers. Full circles mean significant coefficients. (TIFF) [file pone.0096504.s005.tiff]
